# Ecological and evolutionary consequences of alternative sex-change pathways in fish

**DOI:** 10.1038/s41598-017-09298-8

**Published:** 2017-08-22

**Authors:** C. Benvenuto, I. Coscia, J. Chopelet, M. Sala-Bozano, S. Mariani

**Affiliations:** 1 Ecosystems and Environment Research Centre, School of Environment & Life Sciences, University of Salford, Salford, M5 4WT UK; 2 School of Biology and Environmental Science, UCD University College Dublin, Belfield, Dublin, Ireland

## Abstract

Sequentially hermaphroditic fish change sex from male to female (protandry) or vice versa (protogyny), increasing their fitness by becoming highly fecund females or large dominant males, respectively. These life-history strategies present different social organizations and reproductive modes, from near-random mating in protandry, to aggregate- and harem-spawning in protogyny. Using a combination of theoretical and molecular approaches, we compared variance in reproductive success (*V*_k_*) and effective population sizes (*N*_e_) in several species of sex-changing fish. We observed that, regardless of the direction of sex change, individuals conform to the same overall strategy, producing more offspring and exhibiting greater *V*_k_* in the second sex. However, protogynous species show greater *V*_k_*, especially pronounced in haremic species, resulting in an overall reduction of *N*_e_ compared to protandrous species. Collectively and independently, our results demonstrate that the direction of sex change is a pivotal variable in predicting demographic changes and resilience in sex-changing fish, many of which sustain highly valued and vulnerable fisheries worldwide.

## Introduction

Unique among vertebrates^1^, sex-changing fish develop and reproduce as males first and then grow into highly fecund females (protandry), or reproduce initially as females to later change into large dominant males (protogyny). Sequential hermaphroditism has intrigued evolutionary biologists for decades and a great amount of information has been gathered on sex determination, sex differentiation and the plasticity of sex change in fishes^2–5^. The main theoretical model proposed to explain its adaptive value, the size advantage model^6–8^, predicts that sex change should occur when the reproductive success of an individual depends on its size, but more so for one sex than the other. In this scenario, protandry is favoured over fixed separate sexes (gonochorism) when larger females have higher reproductive value than smaller ones (they can produce more eggs), while protogyny is favoured in situations where size allows dominant males to control the reproductive access to females.

**Figure 1:**
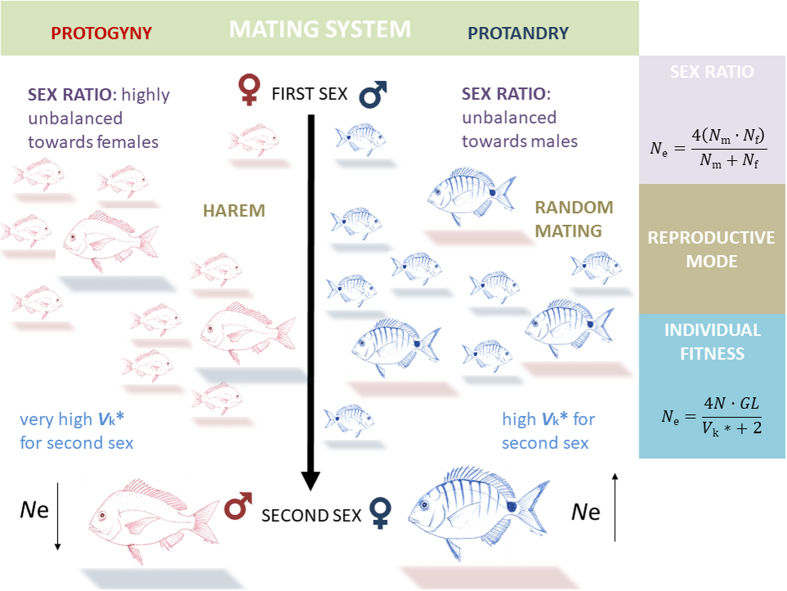
Interplay among individual fitness, life-history traits and population dynamics. Multiple factors affect effective population size (*N*_e_), including many driven by the mating context of the population. The exemplified protogynous species is drawn in red and the protandrous one in blue; shadows indicate the sex of individuals at any point during their lives (red for females; blue for males). In the equations: *N*_m_ = number of adult males; *N*_f_ = number of adult females; *N* = total number of adults in the population; *GL* = generation length; *V*_k_* = variance in individual lifetime reproductive success.

At the population level, we can expect that the variance in individual lifetime reproductive success (*V*_k_*) will influence the demographic trajectory of a population (Fig. 1). Strangely, sex-changing populations have seldom been investigated from a population genetic perspective. In those circumstances the focus has been only on one sex-changing mode, mainly protogyny^9, 10^ or on the comparison between gonochoristic and sex-changing species^11, 12^. Yet, among sequential hermaphrodites, protandry and protogyny stand as two remarkably different life-history strategies as they are shaped by different social systems and reproductive modes^3, 5, 13^. In protandrous species, populations are composed of many small males and fewer large, highly fecund females. The reproductive mode is often monogamy or near-random mating^2, 6^. Protogyny, on the other hand, occurs when there is high potential for polygyny and results in strong social structures dominated by large males^6, 8^, in some cases antagonised by sneakers. Males are territorial and control harems of females or, in group-spawners, larger males are expected to be the most successful. In either case, in protogyny there is a strong sexual selection on males.

Mating system and reproductive mode variations are predicted to influence *V*_k_*^14^ (Fig. 1). In particular, *V*_k_* should be greater in protogyny than in protandry, as large males can monopolize multiple females and small females will tend to choose the larger males, increasing the reproductive success of a small number of larger males.

As a result of such changes over an individual’s lifetime, sequentially hermaphroditic species typically exhibit sex ratios that depart from the balanced ≈ 0.5 observed in gonochoristic species, and are generally skewed towards the ‘first sex’. Such a bias is more pronounced in protogynous species than protandrous ones^15^. Variance in reproductive success and skewed sex ratios are the two most powerful forces that shape a parameter of crucial significance in population genetics, ecology and conservation: the effective population size (*N*_e_), which offers a view of the intensity of genetic drift and the changes in genetic variability in a population and its potential for persistence and resilience^16, 17^. Wright^18^ was the first to realize that the “effective population number” should refer only to the “breeding population and not to the total number of individuals of all ages”; indeed, the effective population number in natural populations is generally smaller than the census size of the same population, sometimes by one or more orders of magnitude.

The direct influence of skewed sex ratio applies mainly to gonochoristic species. In sequential hermaphrodites, the sex ratio is dynamic and many individuals contribute to the coming generations as both sexes (even though not all individuals change sex). Biased sex ratios though reinforce reproductive skew, and higher *V*_k_* values indicate that female-first sex-changers should have lower *N*_e_ than male-first sex-changers (Fig. 1). Thus, the direction of sex change is expected to have an influence on *N*_e_ and, consequently, on population structure.

Calculating *N*_e_ is extremely difficult because it requires a specific knowledge of the demographic and ecological parameters of the natural population under study. As mentioned, *N*_e_ can be influenced by a multitude of factors, including fecundity, birth rate, natural mortality, migration, breeding sex ratio, variance in family size, age-structure, spatial and temporal distributions and reproductive mode^19^. For this reason, direct demographic methods of calculating *N*_e_ are often replaced by indirect genetic methods^20^.

Here, we used a combination of theoretical modelling and molecular genetics to compare the effective population size in several species of protandrous and protogynous fish. A life-history model^21^ was employed to estimate *N*_e_, *V*_k_*, the effective number of breeders per year (*N*_b_), as well as the annual mean number of offspring $(\bar{k})$ and *V*_k_ and *N*_b_ for each sex in two sets of protandrous and protogynous species. At the same time, molecular markers were used to produce empirical ${\hat{N}}_{e}$ estimates for a similar set of protandrous and protogynous species. The combination of these two approaches largely confirms the main expectation of lower *N*_e_ estimates for protogynous than for protandrous species, and collectively unveils previously unrecognised patterns and trends that are of great relevance to the management of marine living resources.

## Results

### Life history modelling

**Figure 2:**
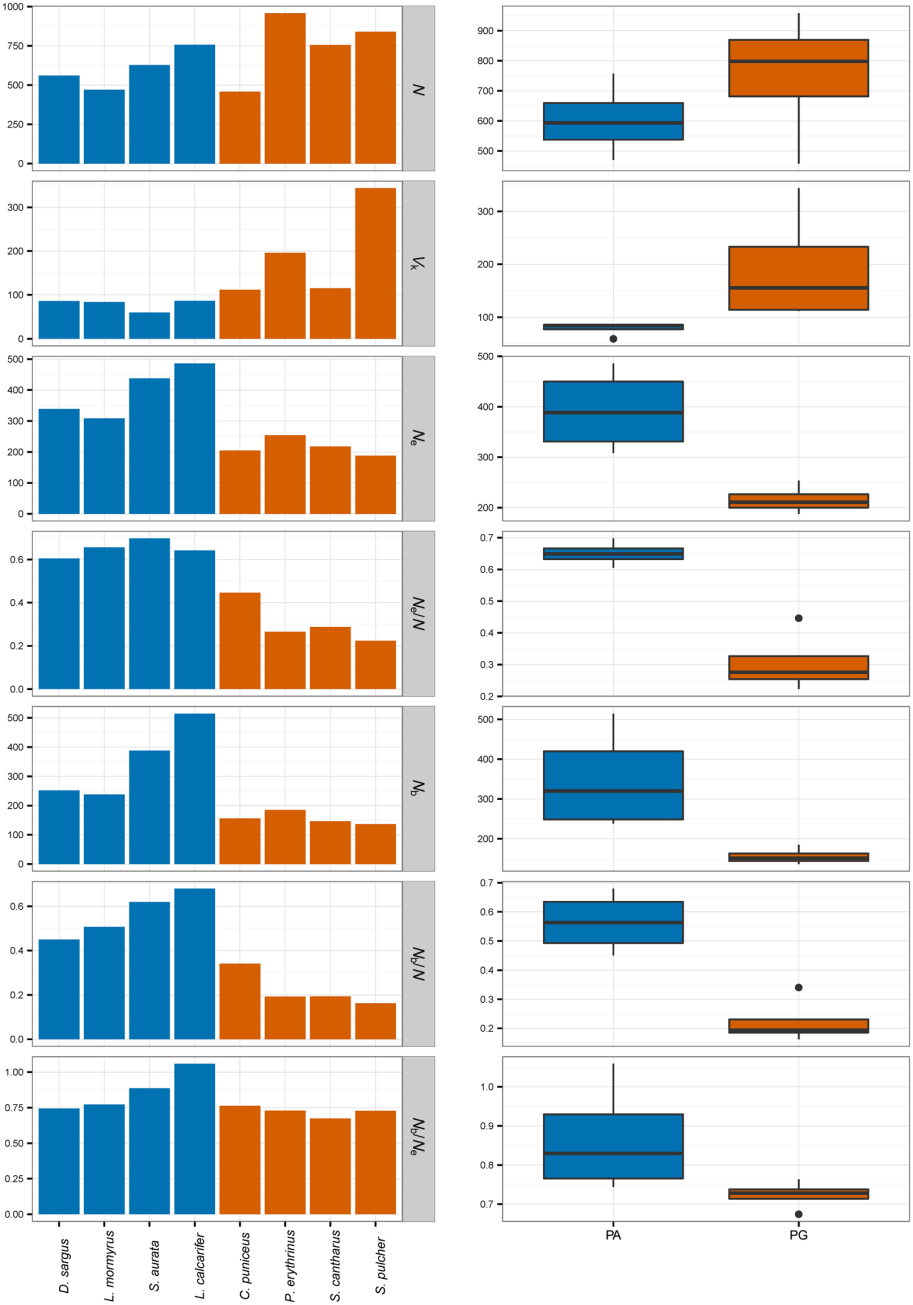
Graphical representation of some key population parameter estimates (obtained with AgeNe) by individual species (panel a) and mating system (panel b); protandry in blue vs. protogyny in red. *N*: estimated number of individuals in the population (census size); *V*_k_*: lifetime variance in reproductive success; *N*_e_: effective population size; *N*_b_: effective number of breeders per year. Key ratios (*N*_e_/*N*, *N*_b_/*N* and *N*_b_/*N*_e_) are also represented.

A range or realistic life-table scenarios were produced, utilizing data from literature on eight species, four protandrous (PA) and four protogynous (PG; Table 1), to estimate^21^ multiple key population parameters (Table 2). As predicted, protogynous species overall showed significantly lower *N*_e_ (mean ± sd: PG = 215.73 ± 28.06; PA = 392.70 ± 83.28; t = 4.03, df = 6, p = 0.006; Fig. 2) and higher lifetime *V*_k_* (mean ± sd: PG = 191.61 ± 108.59; PA = 78.90 ± 12.88; W = 0, p = 0.029; Fig. 2) than protandrous species, in the face of a stable total adult *N* (adult census size) that did not differ significantly between the two groups (mean ± sd: PG = 753.00 ± 213.41; PA = 603.50 ± 120.87; t = −1.22, df = 6, p = 0.280; Fig. 2). Similarly, no significant difference was detected in generation length (mean ± sd: PG = 10.24 ± 5.02; PA = 7.88 ± 1.94; t = −0.87, df = 6, p = 0.416). Indeed, the linear model returned protogyny (t = −7.280; p < 0.001) as the main factor reducing the size effective ratio *N*_e_/*N*, which allows to control for the variance in population abundance estimates (*N*).

**Table 1:** Parameters utilized to construct life history tables.

	Species	Family	Reproductive mode	AgeMax	*L*_*inf*_	*t*_*0*_	*K*	*t*_*m*_	*α*	*β*
Protandry	*Diplodus sargus*	Sparidae	Broadcast spawners	12^63^	45.90	−0.890	0.171	3	0.011	5.10^64^
	*Lithognathus mormyrus*	Sparidae	Broadcast spawners	11^65^	38.44^66^	−1.483^66^	0.200^66^	3	0.010†	5.10^†^
	*Sparus aurata*	Sparidae	Broadcast spawners	12^67^	59.76^67^	−1.711^67^	0.153^67^	2	0.010†	5.10^†^
	*Lates calcarifer*	Latidae	Broadcast spawners	23[32]^68^	143.00	−0.860	0.130	4	27.000^69^	2.89^69^
Protogyny	*Chrysoblephus puniceus*	Sparidae	Haremic	10^12^	44.00^70^	−0.810	0.180	2	10.253^*^	2.60^71^
	*Pagellus erythrinus*	Sparidae	Group spawners	21^31^	41.78^72^	−1.210	0.130	3	0.030^‡^	5.49^‡^
	*Spondyliosoma cantharus*	Sparidae	Haremic and nest guarders	10^73^	47.70	−0.830	0.180	2	0.040^74^^*^	4.60^74*^
	*Semicossyphus pulcher*	Labridae	Haremic and territorial	29^47^	55.77	−0.710	0.126	4	0.00189	5.49^75^

AgeMax: maximum age (in years; in square brackets maximum age recorded for few individuals, not used in the model); *L*_*inf*_: asymptotic length (in cm); *K*: growth coefficient; *t*_*0*_: theoretical age at length = 0; *t*_*m*_: age at first maturity; *α*: constant in the fecundity relationship (adjusted to standard length); *β*: exponent in the fecundity relationship. Values are obtained from FishBase^49^ unless specified.

*Values calculated from available data for *Spondyliosoma cantharus* ^74^; ^†^data obtained for *D. sargus*; ^‡^extrapolated values from fecundity.

**Table 2:** Estimates from AgeNe and used value of age at sex change (*t*_c_) for each species.

	Species	Total *N*	*N*	*GL*	*V*_k_*	*N*_e_	*N*_e_/*N*	*N*_b_	*N*_b_/*N*	*N*_b_/*N*_e_	*t*_c_	♀ $\mathop{k}\limits^{\bar{}}$	♀ *V*_k_	♀ *N*_b_	♂ $\mathop{k}\limits^{\bar{}}$	♂ *V*_k_	♂ *N*_b_
Protandry	*Diplodus sargus*	1875	560	7.449	85.960	338.7	0.605	251.9	0.450	0.744	6	7.650	32.510	91.7	0.573	3.089	201.3
	*Lithognathus mormyrus*	1767	470	6.591	83.526	308.2	0.656	238.2	0.507	0.773	6	9.942	37.910	78.3	0.600	2.651	248.7
	*Sparus aurata*	1957	627	6.751	59.668	437.9	0.698	388.2	0.619	0.887	6	6.592	13.110	131.8	0.554	1.751	368.0
	*Lates calcarifer*	2356	757	10.745	86.435	486.0	0.642	514.5	0.680	1.059	8	4.508	7.282	195.0	0.469	1.487	378.0
Protogyny	*Chrysoblephus puniceus*	1755	458	5.810	111.782	204.2	0.446	155.9	0.340	0.763	5	0.588	1.844	366.9	18.170	18.170	43.6
	*Pagellus erythrinus*	2326	958	12.564	196.089	253.7	0.265	185.0	0.193	0.729	9	0.443	1.733	297.7	14.610	67.680	54.8
	*Spondyliosoma cantharus*	1756	756	6.352	114.935	217.3	0.287	146.5	0.194	0.674	5	0.592	2.435	269.5	15.120	143.000	42.4
	*Semicossyphus pulcher*	2455	840	16.219	343.616	187.7	0.223	136.5	0.163	0.727	13	0.415	1.619	301.0	23.410	83.32.0	38.5

Total *N*: estimated number of individuals in the population; *N*: estimated number of adult individuals (adult census size); *GL*: estimated generation length; *V*_k_*: lifetime variance in reproductive success; *N*_e_: effective population size; *N*_b_: effective number of breeders per year; $\mathop{k}\limits^{\bar{}}$: annual mean number of offspring; ♀: female; ♂: male. The last six columns report $\mathop{k}\limits^{\bar{}}$, *V*_k_ and *N*_b_, per sex, per breeding cycle.

As all the species under study are iteroparous, *N*_b_ (effective number of breeders per breeding cycle) was also calculated and found to be significantly lower in protogynous than in protandrous species (mean ± sd: PG = 155.98 ± 20.91; PA = 348.20 ± 129.91; W = 16, p = 0.0286; Fig. 2). The linear model returned protogyny (t = −6.179, p = 0.003) as the main factor reducing *N*_b_/*N* (again, to control for the variance in population abundance estimates, *N*), with maximum length as a covariate, which significantly increased this size effective ratio (t = 2.954, p = 0.042).

The analysis of the *N*_b_/*N*_e_ ratio revealed no influence of the mating system (t = −2.074; p = 0.093), but a significant effect of maximum length (t = 4.886; p = 0.004). The male-first sex-changer *L. calcarifer* showed a ratio larger than 1 (*N*_b_/*N*_e_ = 1.059), meaning that in this species the number of breeders per year is actually higher than the overall effective population size (Fig. 2).

**Figure 3:**
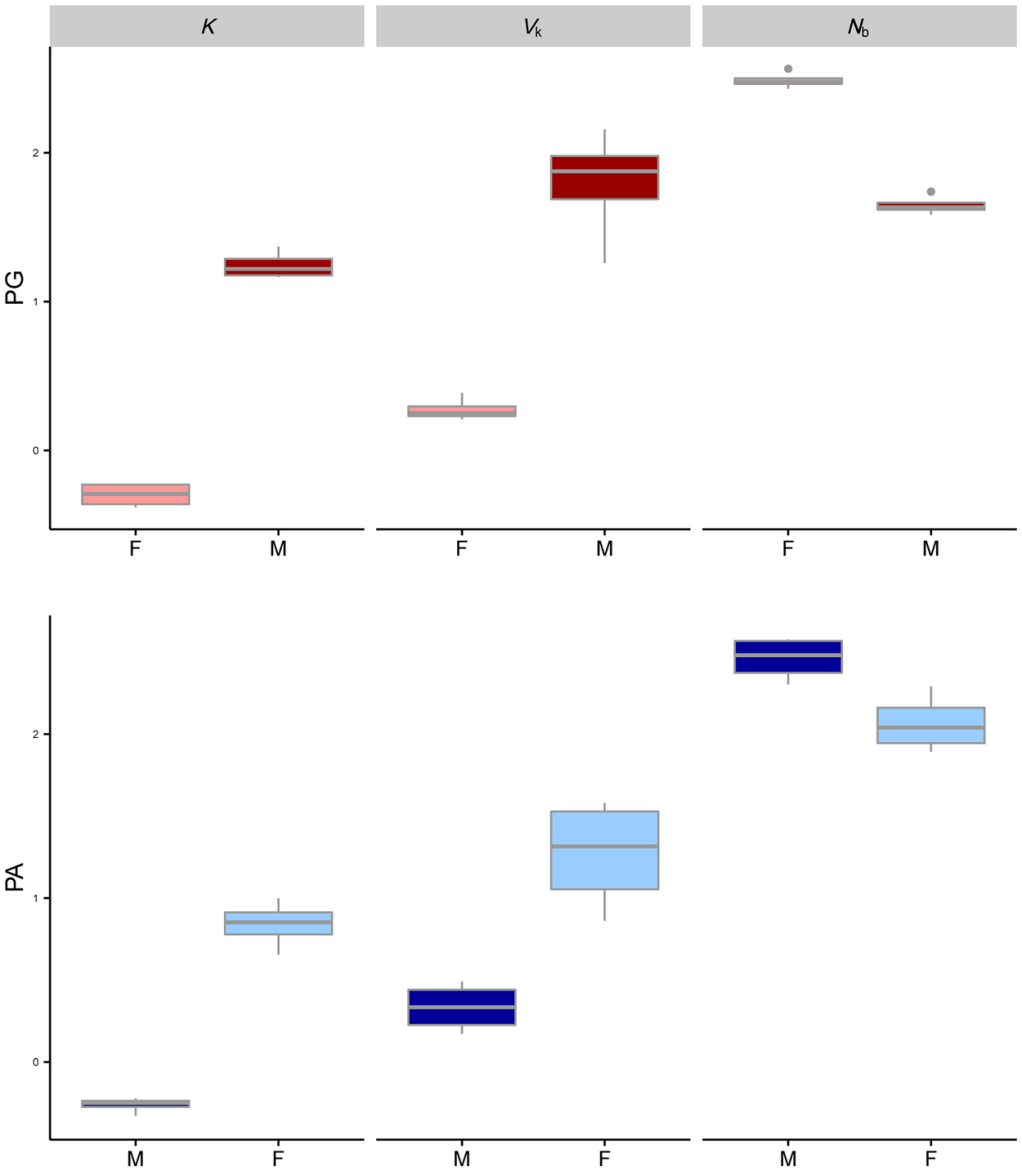
Graphical representation of some annual key parameter estimates (obtained with AgeNe) in each life history strategy, per sex. $\bar{k}$: annual mean number of offspring; *V*_k_: annual variance in reproductive success; *N*_b_: annual effective number of breeders. Values are log_10_ transformed to improve visualization. Protandry in blue (panel a); protogyny in red (panel b); lighter colour: females - F; darker colour: males - M.

It was also possible to analyse $\bar{k}$, and annual *V*_k_ and *N*_b_ for each sex. Graphically (Fig. 3) it is easy to visualize the reproductive strategy for an individual when it reproduces as a different sex during its lifetime, changing from the first sex (male in protandry and female in protogyny) to the second sex (female in protandry and male in protogyny). Individuals belonging to protandrous species have higher annual $\bar{k}$ (mean ± sd: ♀** = **7.17 ± 2.26; ♂ = 0.55 ± 0.06; = 5.06, df = 6, p = 0.002) and *V*_k_ (♀ = 22.70 ± 14.80; ♂ = 2.24 ± 0.75; t = 5.06, df = 6, p = 0.002) when they are older and larger (second sex, female, Fig. 3a). For this latter stage of life (the female phase), in the population there are fewer breeders per year than for the male phase (*N*_b_♀ = 124.20 ± 52.39; *N*_b_ ♂ = 299.00 ± 87.71; t = −3.561, df = 6, p = 0.012). A mirrored situation applies to protogynous species, and in this case the magnitude is even stronger (Fig. 3b): larger males second sex) have much higher annual $\bar{k}$ (♂ = 17.82 ± 4.04; ♀ = 0.51 ± 0.09; Welch t-test t = −24.892, df = 5.87, p < 0.0001), higher *V*_k_ (♂ = 78.03 ± 51.43; ♀ = 1.91 ± 0.36; W = 0, p = 0.029), and lower *N*_b_ than smaller females, the first sex (♂ = 44.83 ± 7.00; ♀ = 308.78 ± 41.25; W = 16, p = 0.029). The even greater mismatch between the number of breeders for the two sexes reflects nicely the description of haremic species, where few males monopolize the majority of females.

**Figure 4:**
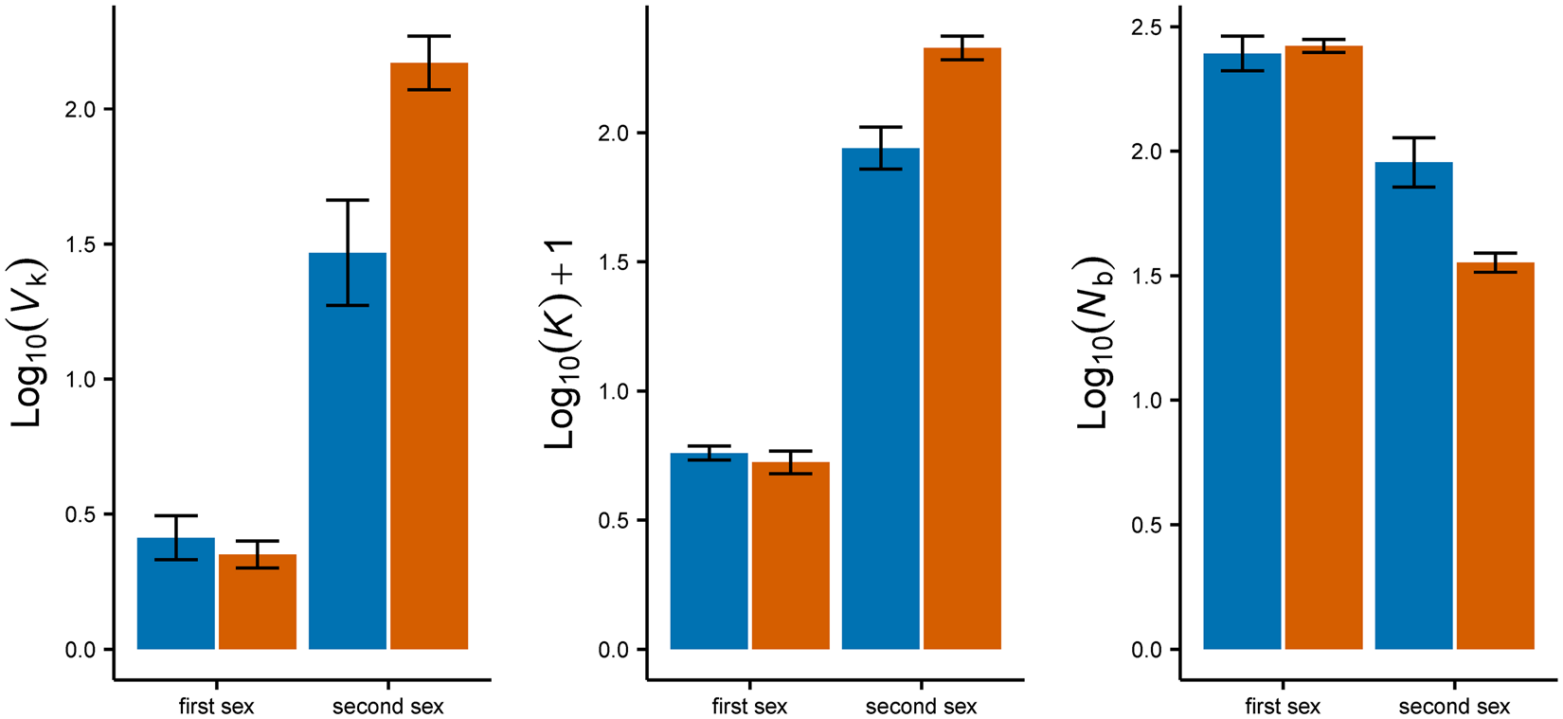
Interaction plots of some annual key parameter estimates (obtained with AgeNe) in each life history strategy per sex. $\bar{k}$: annual mean number of offspring; *V*_k_: annual variance in reproductive success; *N*_b_: annual effective number of breeders. Values are log_10_ transformed to improve visualization. Protandry in blue; protogyny in red.

In general, the trend is similar between protandry and protogyny: the second sex has always higher $\bar{k}$ and *V*_k_ (which confirms the individual advantage of a change of sex in both systems) and lower *N*_b_ than the first sex (Fig. 3). When comparing the reproductive success of first and second sex across the two systems, it is interesting to see a significant interaction (mating system [PA, PG] crossed with sequential sex [first, second]: t = 26.06; P < 0.0001; Fig. 4): the second sex is comparatively more successful in protogyny, whereas the first sex is slightly more successful in protandry. Protogynous male breeders (second sex in red in Fig. 4) are more successful (but less numerous) than protandrous females (second sex in blue in Fig. 4) but protogynous females (first sex in red in Fig. 4) are not more successful than protandrous males (first sex in blue in Fig. 4), even though at this stage the fitness difference between the first and second sex is not as large as it will become later in life, after sex change.

### Genetic evidence

**Figure 5:**
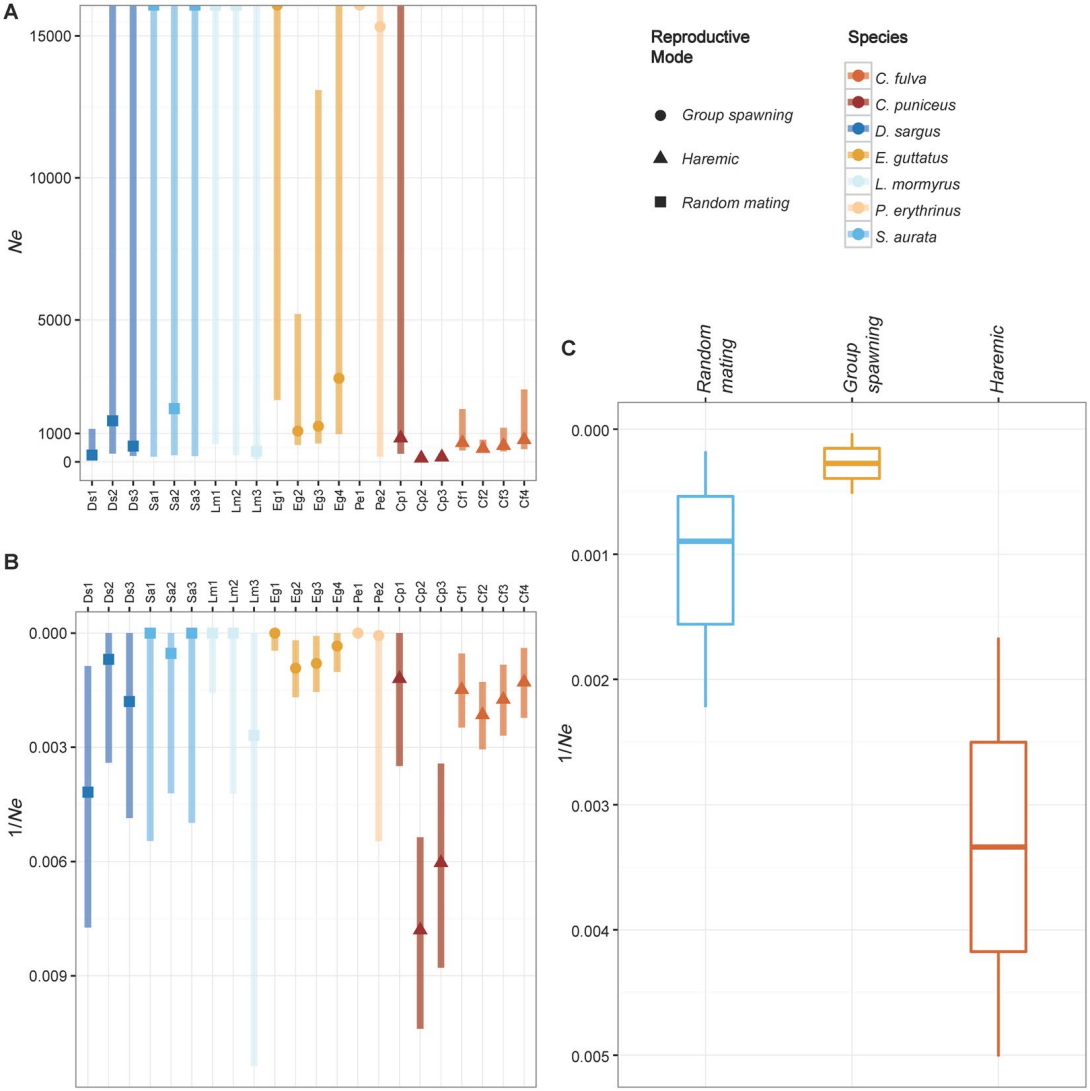
Effective population size (${\hat{N}}_{e}$) estimates, obtained with LDNe for multiple populations of the seven species under study. Bars in the tones of blue: protandrous species; bars in the tones of orange: protogynous species. (**A**) ${\hat{N}}_{e}$ values (up to infinite); (**B**) $1/{\hat{N}}_{e}$ (note the reverse y axis as an aid to visualize ${\hat{N}}_{e}$ values - higher $1/{\hat{N}}_{e}$ values imply lower ${\hat{N}}_{e}$); (**C**) $1/{\hat{N}}_{e}$ (reverse y axis) by reproductive mode.

We had generated genotypic data from five sparid species: three protandrous, *Diplodus sargus*, *Lithognathus mormyrus* ^22^ and *Sparus aurata* ^23^, and two protogynous, *Chrysoblephus puniceus* ^12^ and *Pagellus erythrinus* ^24^. Furthermore, two more protogynous species, *Cephalopholis fulva* and *Epinephelus guttatus*, were included in the analyses, based on datasets made available by Portnoy and colleagues^9^. No significant signals of large allele drop out, scoring error were detected across each species. Only *C. puniceus* had one marker (SL35) that had a strong probability for null alleles, and was hence removed from the statistical analysis. Average observed (*﻿H﻿*_o_)﻿ and expected (﻿*H*_e_)﻿ heterozygosities were respectively 0.756 and 0.823 for *D. sargus*; 0.844 and 0.836 for *L. mormyrus*; 0.768 and 0.776 for *S. aurata*; 0.532 and 0.534 for *C. fulva*; 0.701 and 0.860 for *P. erythrinus*; 0.794 and 0.829 for *C*. *puniceus* and 0.690 and 0.694 for *E. guttatus* (Table 3; see Table S1 for genetic diversity parameters by locus and by population). Two protogynous species, *P. erythrinus* and to a lesser extent *C. fulva*, showed signs of heterozygotes deficiency, with values of *H*_o_ smaller than *H*_e_. This translates into high and positive *F*_is_ values recorded especially for *P. erythrinus*. Overall, *H*_o_ was on average higher for the protandrous than the protogynous species (W = 99, p = 0.007; Fig. S1), while no significant difference was found for *H*_e_ (W = 79.5, p = 0.171) or *A*_R_ (W = 43, p = 0.324). Population structure was also investigated using *F*_st_, the Bayesian clustering implemented in STRUCTURE (Fig. S2) and the Discriminant Analysis of Principal Component, DAPC (Fig. S3; Fig. S4). Although some species did show low but significant pairwise *F*_st_ values, overall only *L. mormyrus* revealed the presence of substantial population structure, with both analyses. This was expected as the dataset contains Atlantic and Mediterranean populations, which are separated by a strong phylogeographic break^22^. *Cephalopholis fulva* and *S. aurata* also showed some signal of low but significant differentiation with pairwise *F*_st_ (Table S2), although STRUCTURE failed to identify any sub-structuring. We decided to estimate ${\hat{N}}_{e}$ per single population/location, rather than pooling them together. Estimates of ${\hat{N}}_{e}$ calculated with LDNe, varied between 128 (Cp2) and infinite (Lm1, Lm2, Sa1, Sa3, Pe1, Eg1; Table 3; Fig. 5a). Given the high skew towards large values (i.e., infinite estimates), we calculated also $1/{\hat{N}}_{e}$ (Fig. 5b). Overall, we did not detect any significant difference when we compared $1/{\hat{N}}_{e}$ across all the populations classified by sex-changing system (protogyny *vs*. protandry: W = 44, p = 0.345), but, given the high variance (Fig. 5c) recorded for protogynous species, we repeated the analysis using their reproductive mode, and found that haremic species stand out as having significant higher $1/{\hat{N}}_{e}$ than the other species (random mating *vs*. group spawning *vs*. harem: Kruskall-Wallis χ^2^ = 8.0101, df = 2, p = 0.018).

**Table 3:** Genetic diversity parameters (n = sample size; loci = number of microsatellite loci) for multiple populations (with relative ID) of each species.

	Species	Population	ID	n	loci	*H*_o_	*H*_e_	*F*_is_	*A*_R_	${\hat{N}}_{e}$
Protandry	*Diplodus sargus*	France – Sète Italy – Livorno Croatia – Rovinj	Ds1 Ds2 Ds3	49 49 50	10 10 10	0.738 0.754 0.776	0.822 0.826 0.831	0.121 0.089 0.061	15.094 15.222 14.428	239.5 (129.3–1155.7) 1447.1 (293.7–∞) 556.3 (205.9–∞)
	*Lithognathus mormyrus*	Spain – Cadiz Italy – Sabaudia Croatia – Duce	Lm1 Lm2 Lm3	70 22 26	9 9 9	0.873 0.838 0.821	0.869 0.828 0.812	−0.007 −0.016 −0.008	14.576 12.333 10.455	∞ (636.5–∞) ∞ (237.6–∞) 372 (88–∞)
	*Sparus aurata*	France – Ile d’Oleron Portugal – Aveiro Spain – Cadiz	Sa1 Sa2 Sa3	40 50 34	12 12 12	0.755 0.778 0.772	0.774 0.768 0.785	0.016 −0.002 0.017	7.829 8.421 7.911	∞ (183.2–∞) 1875.5 (237.8–∞) ∞ (200.8–∞)
Protogyny	*Chrysoblephus puniceus*	South Africa - Port Edward South Africa - Park Rynie South Africa - Richards Bay	Cp1 Cp2 Cp3	43 43 39	11 11 11	0.782 0.810 0.790	0.827 0.831 0.830	0.048 0.025 0.040	18.005 18.161 18.00	834.9 (286.7–∞) 128.2 (96.3–186.5) 165.8 (113.8–291.6)
	*Pagellus erythrinus*	France – Sète Italy - Livorno	Pe1 Pe2	48 48	9 9	0.700 0.701	0.843 0.877	0.145 0.188	10.556 10.989	∞ 15325.4 (183.1–∞)
	*Cephalopholis fulva*	U.S. Virgin Islands - St. Thomas U.S. Virgin Islands - St. Croix Puerto Rico - West coast Puerto Rico - East coast	Cf1 Cf2 Cf3 Cf4	106 110 100 100	24 24 24 24	0.516 0.538 0.546 0.526	0.523 0.537 0.550 0.527	0.022 0.003 0.014 0.000	9.194 9.660 9.552 9.547	673.2 (402.9–1855.9) 465.3 (327.7–777.6) 574.1 (371.3–1199.5) 776.2 (449.4–2546.1)
	*Epinephelus guttatus*	U.S. Virgin Islands - St. Thomas U.S. Virgin Islands - St. Croix Puerto Rico - West coast Puerto Rico - East coast	Eg1 Eg2 Eg3 Eg4	101 101 95 100	19 19 19 19	0.684 0.682 0.701 0.691	0.693 0.692 0.697 0.694	0.006 0.010 −0.009 0.006	14.697 15.093 14.526 14.983	∞ (2179.4–∞) 1083.5 (594.3–5209) 1254.1 (647.3–13093.9) 2944.2 (979.7–∞)

*H*_o_ = observed heterozygosity; *H*_e_ = expected heterozygosity; *F*_is_ = coefficient of inbreeding; *A*_R_ = allelic richness. Effective population size (${\hat{N}}_{e}$) estimated with LDNe.

## Discussion

Sequentially hermaphroditic fish are an intriguing group of animals. Their life history includes, for the majority of the individuals in a population (the exception being primary females in protandry and primary males in protogyny), a complete rearrangement of gonads and behaviours which allow them to reproduce as the opposite sex, at a certain stage of their life^2^.

While fecundity and reproductive success are expected to increase with size for both females and males, under certain circumstances (mainly related to mating context^3, 5^), the fitness advantage can increase more rapidly for one sex than the other. In this case, it is beneficial to match the sex with the higher reproductive value at a given size, in order to maximise the reproductive output^6–8^. Since the sexual-transition is mating-system dependent, there is not a single direction to change sex, but two, from male to females or *vice-versa*; in our study we have not considered bidirectional sex-changers^25^ given their low occurrence.

To our knowledge, no studies have attempted to calculate and compare the fitness of each sex stage in both sequentially hermaphroditic models (protandry and protogyny). Using a theoretical model based on life history traits, we were able to compare sex-specific reproductive outputs in eight sex-changing species (four protandrous and four protogynous), and their corresponding annual variance. As predicted by theory, in both systems (protandry and protogyny) the second sex is more successful than the first one in terms of the annual average number of offspring, $\bar{k}$ and experiences higher variance in reproductive success, *V*_k_. Also, in both cases, the second sex is composed by a smaller number of annual breeders (*N*_b_) compared to the first sex (Fig. 3). The magnitude of these values is different between the two systems and this reflects the underlying reproductive systems that characterize them: in protogyny, sex-changed males (mostly haremic) face a large increase in $\bar{k}$ and *V*_k_ compared to their previous female stage (and there are less male breeders: they are few and dominant), much larger than the increase occurring during a protandrous transition (Fig. 3).

Recently there has been an increased emphasis on the connections between individual life history traits and population-level responses^26, 27^ which results in a link between demographic and evolutionary processes^28^. Lifetime *V*_k_* can be translated to the population level, which allows us to address a broadly relevant question, especially in light of conservation and management of fish stocks and biodiversity: are the population trajectories of protandrous and protogynous species different? Our estimates confirmed that the *V*_k_* of protogynous species is significantly higher than the *V*_k_* of protandrous ones (as hypothesised: many protogynous haremic species have few larger males who can successfully fertilize the majority of the females; Fig. 3). As a consequence, we found that protogynous species have significantly lower *N*_e_ than protandrous ones (while having similar number of adults in the population), thus supporting the initial hypothesis that the direction of sex change plays an important role in shaping the demographic trajectories of fish population under natural mortality (our life history tables did not include fishing pressure).

For iteroparous species, it has been suggested to estimate the effective number of breeders per reproductive cycle (*N*_b_), which is easier to calculate than the effective population size^28, 29^. The ability to change sex adds an extra level of complexity to the analysis of *N*_e_ in organisms which grow indefinitely, reproduce multiple times in their lives and have overlapping generations: in this case the focus on *N*_b_ is more relevant than the number of reproducing individuals per generation (*N*_e_), as the same individual in different years will reproduce using a different strategy. We thus calculated overall *N*_b_ estimates and a series of key effective size ratios. By definition, protogynous species have highly skewed sex ratios (unbalanced towards the first sex), which should result in low numbers of male successful breeders (*N*_b_). Also, *N*_e_/*N* ratios are expected to be low in species with high fecundity but high *V*_k_*^30^ and *N*_e_/*N* should approximately be similar to *N*_b_/*N* ratios^30^. All these theoretical expectations were confirmed in our analyses: *N*_b_ values, as well as *N*_e_/*N* and *N*_b_/*N* ratios were found to be significantly lower in protogynous than in protandrous species. We did not detect differences in *N*_b_/*N*_e_ in the two groups, but in one case, the protandrous barramundi (*L. calcarifer*), *N*_b_/*N*_e_ exceeded 1. According to theoretical and empirical estimates, a long life span leads to higher values of *N*_b_ and delayed maturation leads to an increase of *N*_e_^28^_._ The barramundi is very long lived but has an early maturation, which explains the somewhat paradoxical (yet not unique^28^) value obtained.

We were able to strengthen our understanding of the consequences of changing sex in opposite directions by testing the central hypothesis (protogynous species have lower *N*_e_ than protandrous species) also using independent molecular datasets on multiple populations of five sparid species: higher ${\hat{N}}_{e}$ estimates were obtained for the protandrous species (with the exception of one population of *D. sargus*), but not all the estimates from the two protogynous species available (which do not fully correspond to the species used for the life-history modelling) conformed to the expectation of low ${\hat{N}}_{e}$ values: indeed, while *C. puniceus* (with a lek-like system where males defend territories) presented low ${\hat{N}}_{e}$ for the majority of the populations, both populations of *P. erythrinus* presented high value of ${\hat{N}}_{e}$, comparable with the ones obtained in protandrous species (Fig. 4; Table 3). Even with estimates obtained from life history tables, the *N*_e_ estimate for this species was higher than the other species under consideration (Fig. 2; Table 2). *Pagellus erythrinus* are group-spawners: their *V*_k_* is not as high as haremic species, but a protogynous sex change is still advantageous in this case: being a large male can be beneficial in the spawning area^31, 32^, where the lack of haremic structure removes the typically reduced level of sperm-competition which is the norm in protogynous species^33^. Furthermore, the pronouncedly lower-than expected *V*_k_ in *P. erythrinus*, may to some extent stem from increased levels of female fecundity and/or $\mathop{k}\limits^{\bar{}}$, or a possible unexplored greater contribution of primary males^34^, which the standardised approach we employed (see Methods) would not be able to account for.

To test the possible interplay between sex-changing system and reproductive mode, we used two more datasets available for two protogynous groupers, the group-spawner *E. guttatus* and the haremic *C. fulva* ^9^: indeed, the former exhibited values similar to *P. erythrinus*, while the latter showed estimates comparable to *C. puniceus*, thereby revealing an additional layer of complexity to the scenario: we detected a decrease of ${\hat{N}}_{e}$ along a gradient of reproductive modes (Fig. 4) and found that harem-spawners protogynous species have lower ${\hat{N}}_{e}$ than group-spawning ones. Thus, behavioural traits contribute to a fuller understanding of the patterns of *N*_e_ and *V*_k_* in sequential hermaphrodites.

Overall, the estimates from life history tables confirmed the theoretical expectation that, in general, regardless of the direction of sex change, individuals use the same overall strategy, producing less offspring $(\bar{k})$ with less variance in reproductive success (*V*_k_) as the first sex and more offspring with higher *V*_k_ as the second sex, later in life. Concurrently, at the population level, the majority of breeders (*N*_b_) reproduce as the first sex. Although this trend is consistent in the two mating strategies (protandry and protogyny), its magnitude is much higher in protogynous species, resulting in a stronger reduction of *N*_e_ at the population level. Thus, the direction of sex change has an influence on the overall *N*_e_ of the populations and combining all sex-changing fish species in one category is ultimately incorrect and can be misleading for conservation and management practices.

A second independent analysis, based on molecular data also indicated that a protogynous mating system is prone to more significant reductions of ${\hat{N}}_{e}$ than a protandrous system. Moreover, this second analysis uncovered the fact that, in protogynous populations, *V*_k_* may also be dependent on reproductive modes (from aggregate- to harem-spawning) and ${\hat{N}}_{e}$ can change accordingly. The comparison, within protogynous sex-changing modes, between group-spawners and haremic species confirms the importance of considering and assessing the influence of mating systems and reproductive modes, in order to understand the demography of populations^35^. The possibility that several natural stocks of haremic protogynous species may owe their persistence to a disproportionately small number of extremely successful old males fits with recent concerns regarding many valuable warm-water fisheries worldwide^36^, and calls for the practical consideration, not only of sex change, but also of its direction and the behavioural structure underpinning it, in the management arena. Future analyses should focus on the evaluation of the consequences of increased mortality by fishing pressure and the populations’ compensatory capacities^37^. Following this path can significantly expand our ability to predict the viability of many commercially exploited reef-associated species in tropical and warm-temperate areas, and enable us to do something useful for their preservation.

## Materials and Methods

### Choice of species

Four protandrous and four protogynous species were selected (Table 1), based on existing knowledge of their biology, to produce realistic life history tables. Six of them belong to the Sparidae, a very heterogeneous family in terms of reproductive styles^38^, which includes both protandrous and protogynous species. We previously generated genetic data for five out of these six sparid species, which we used in our molecular analysis (Table 3), in the attempt to minimize the biases that may arise comparing phylogenetically distant taxa. Two more protogynous species^9^, characterized by different social structures (aggregate- and harem-spawners) were also included in the analysis.

### Theoretical modelling

Key life history parameters (Table 1) were retrieved from the literature and fed into a demographic model^21^, implemented in the freely available software AgeNe, version 2.0 (http://conserver.iugo-cafe.org/node/2876). For each species, Leslie matrices were built and age-specific survival (*s*_x_), fecundity and sex ratios were calculated.

#### Age-specific survival rates

Natural mortalities (*m*) were calculated for length classes following Charnov and colleagues^39^

1 $$m=[{(L/{L}_{inf})}^{-1.5}]\,\cdot K$$

where *L*_inf_ is the asymptotic length (in cm), *L* is the fish standard length (in cm) and *K* is the growth coefficient of the von Bertalanffy equation:

2 $${L}_{x}={L}_{inf}(1-{e}^{(-K(x-x{}_{0}))})$$

where *L*_*x*_ is the fish total length (in cm) at age *x*, and *x*_0_ is the theoretical age at length = 0. Length classes were assigned to age classes (eq. 2) and length values for each class were compared with known data from the literature, to ensure realistic estimates. Natural instantaneous mortalities were then transformed in annual mortality rates per year (d^40^) using the formula:

3 $$d=1-{e}^{-m}$$

To prevent survivorship in the oldest age class, age-specific survival rates were calculated as:

4 $${s}_{x}={s}_{A}-(0.5/x)-\{[{e}^{(x-s{}_{A})}]\cdot {s}_{A}-(0.5/x)\}$$

which is based on a generally accepted type III survivorship curve, where *s*_*A*_ is the asymptotic maximum survival (*s* = 1 − *d* = e^−*m*^, from eq. 3) and *x* is the age. This introduced senescence reduces the adult population to zero at the maximum age (as reported in the literature). Equation 4 adjusts survival for age, following the rationale that natural mortality increases exponentially with age^24, 41^. Thus, survival increases with size until at a certain age and then decreases in older fish till all fish are dead in the oldest age class. Based on greater availability of bibliographic information, combined data for the two sexes were used. Even though this is a simplification^42^, the assumption of size-dependent (and not sex-dependent) survival rates should not significantly alter the results. Indeed, for the majority of individuals (excluding the ones which do not change sex, i.e., primary males in protogyny and primary females in protandry) sex is correlated with size. This means that male survival can be assumed to be the same as female survival, but this value changes with the size of the fish^39, 43^. Average mortality data (across all size classes) were compared to known mortality rates from literature, to ensure realistic estimates (Table S3).

#### Age-specific fecundity

Female fecundity can be calculated with an allometric (power function) relationship^44–46^

5 $${\rm{Female}}\,{\rm{fecundity}}({N}_{eggs})=\alpha {L}^{\beta }$$

where *α* and *β* are constant. Surprisingly, not many data on fecundity are available from the literature. We used the data available and extrapolated the missing data, comparing calculated ranges of fertility with known ranges from literature (when available), to ensure realistic estimates (Table S3). From a practical standpoint (count of gametes), it is more difficult to calculate male fecundity. Following Alonzo and Mangel^45, 46^ we considered male fecundity as 1000 times female fecundity.

#### Age-specific sex ratio in the population

The sex ratio in the population changes with the size of the individuals^46^ and thus it is different among age-classes. Individuals change sex at a specific age (*t*_c_). The proportion of the terminal sex (*S*; males in protogynous species and females in protandrous species) was calculated with the formula:

6 $$S={S}_{i}+({S}_{f}-{S}_{i})/\{1+{e}^{[-(t-{t}_{c})]}\}$$

where *S*_i_ is the proportion of non sex-changing individuals (primary males in protogynous species and primary females in protandrous species) and *S*_f_ is the final proportion of the terminal sex (overall sex ratios for each species are reported in Table S3).

Given these age-specific parameters (age-specific survival rate *s*_x_; birth rate *b*_x_ and sex ratio), AgeNe can produce complete life history tables for each sex, with estimates of total number of individuals in the population (total *N*) and estimated number of adults (adult census size *N*), generation length (*GL* i.e., the average age of parents of a newborn cohort, in years), lifetime *V*_k_*, *N*_e_, and annual number of breeders (*N*_b_). Moreover, the software estimates annual *V*_k_, annual mean number of offspring ($\bar{k}$) and annual *N*_b_ for each sex. We searched published literature to obtain realistic parameters for our models. We are aware that a great deal of local variation exists in these parameters^47, 48^ and we acknowledge that geographically targeted predictions should be made for individual populations. Here, we used data from FishBase^49^ to insure general (and not locality-specific) estimates (Table 1). We checked that our life tables reproduced realistic scenarios and we made changes only when recent articles reported higher maximum length than originally reported in FishBase; Table S3). Our goal was to obtain a general (and not local) estimate of *N*_e_, for comparative purposes among species characterized by different mating systems.

### Molecular analysis

Samples were genotyped using microsatellite loci (Table S4) run on ABI 3130xl Genetic Analyzer capillary sequencers (Applied Biosystems©). Markers were amplified using fluorescent primers labelled with 6-FAM, NED, PET and VIC dyes (Applied Biosystems©). For *S. aurata*, we used 12 of the 15 loci used in the original study^23^, since we removed three possible candidates for directional or balancing selection. Data were analysed with MICRO-CHECKER^50^ to check for large allele drop out and scoring errors; frequencies of null alleles were estimated using the software FreeNA^51^. We retained loci with moderate frequency (0.05 < r < 0.20) of null alleles^51^. Estimates of genetic diversity, including expected (*H*_e_) and observed (*H*_o_) heterozygosities, allelic richness (*A*_R_) and coefficient of inbreeding (*F*_is_), to test for departure from Hardy-Weinberg equilibrium, were calculated using the package hierfstat ^52^ while pairwise *F*_st_ values and relative 99% confidence intervals were calculated using the package DiveRsity^53^ both developed in R^54^. We also checked for the presence of population sub-structuring using two different approaches: STRUCTURE 2.3.4^55, 56^ and the Discriminant Analysis of Principal Component (DAPC) as performed in the R^54^ package ADEGENET v 2.0.2^57, 58^. Only adult animals were used in the analyses. When data from more than three populations of sparids from our lab were available, we chose truly protandrous and protogynous populations (based on logistic models for size at sex change), i.e., populations with reduced frequencies of non sex-changing individuals (primary males and primary females). Molecular markers were used to calculate *N*_e_ estimates using the software LDNe^59^, as implemented in NeEstimator V2^60^ following Gilbert and Whitlock^61^. Rare alleles with frequencies lower than 0.02 were excluded from our calculations, as suggested by Waples and Do^62^.

### Statistical analyses

Comparisons between protandrous and protogynous species were performed with Student’s t-test, Welch t-test, Wilcoxon and Kruskall-Wallis tests depending on the normality and equality of variance of the data. We assessed the effect of mating system (PA and PG) on the effective size ratio *N*_e_/*N* and *N*_b_/*N* (to control for abundance), using a series of covariates (after checking for multicollinearity; Fig. S5). For *N*_e_/*N* the best linear model (Multiple R^2^ = 0.9396, F_2,5_ = 38.92; p = 0.0009; AIC = −18.84) included generation length as a covariate; for *N*_b_/*N* (Multiple R^2^ = 0.9446, F_3,4_ = 22.73, p = 0.006), the significant covariate was maximum length. A linear model was also run for the ratio *N*_b_/*N*_e_ (R^2^ = 0.8929, F_2,5_ = 0.85, p = 0.004). Linear models were fitted to annual *V*_k_, $\bar{k}$ and *N*_b_ data considering mating system and sequential sex (first vs second, regardless if male or female) as interaction terms. All analyses and graphical output were performed using the freely available scripts within the R environment^54^.

### Ethical statement

Fish samples were purchased from local fishermen. All methods and protocols were carried out in accordance with relevant guidelines and regulations. Ethical approval AREC-P-10–23 was obtained from UCD, University College Dublin.

### Data and materials availability

Microsatellite genotypes can be found on Dryad Digital Repository - doi:10.5061/dryad.n722g4d3 for *S. aurata* ^23^; doi:10.5061/dryad.g36ch for *C. puniceus* ^12^; doi:10.5061/dryad.sj894 for *E. guttatus* and *C. fulva* ^9^; genotypes for *D. sargus*, *P. erythrinus* and *L. mormyrus* for will be made available upon acceptance.

## Electronic supplementary material

Supplementary materials
