# Supplementary material for: Ecological and evolutionary consequences of alternative sex-change pathways in fish
Source: Sci Rep. 2017 Aug 22;7:9084. doi: 10.1038/s41598-017-09298-8 (PMC5567342; doi:10.1038/s41598-017-09298-8)
Supplement: Supplementary file 1 — Supplementary materials [file 41598_2017_9298_MOESM1_ESM.pdf]

Supplementary Materials

Ecological and evolutionary consequences of alternative sex-change pathways in fish

C. Benvenuto, I. Coscia, J. Chopelet, M. Sala-Bozano, S. Mariani

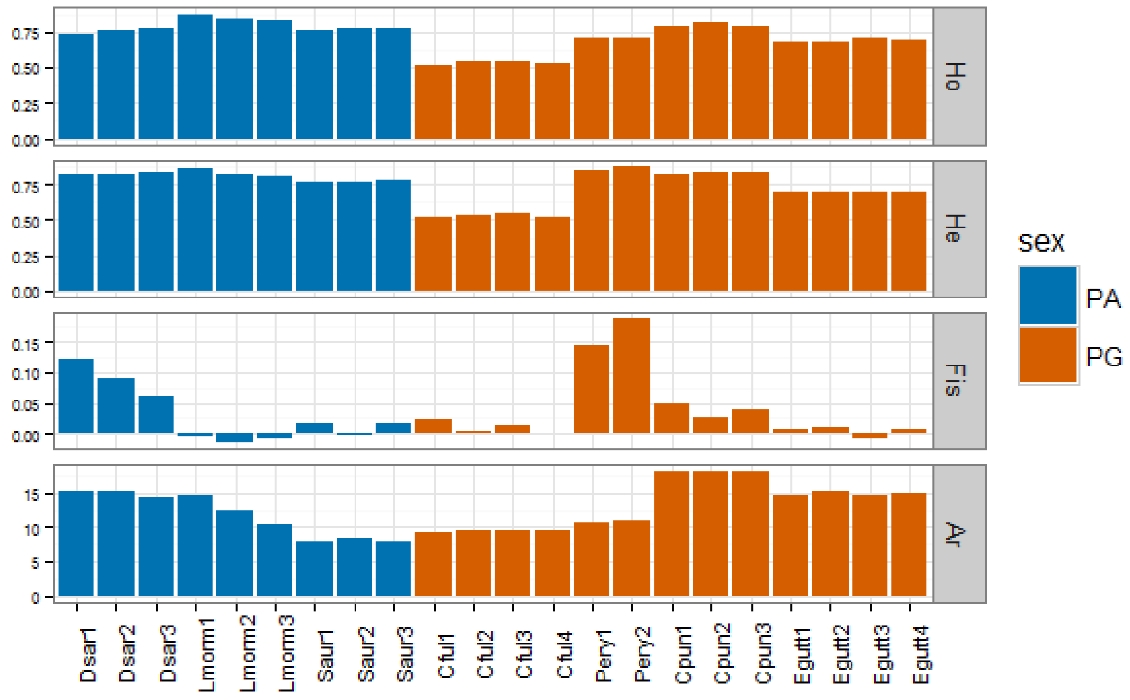

Figure S1. Estimates of observed (H_o) and expected (H_e) heterozygosities, coefficient of inbreeding (F_{is}) and allelic richness (A_R) for each population of the seven species under investigation.

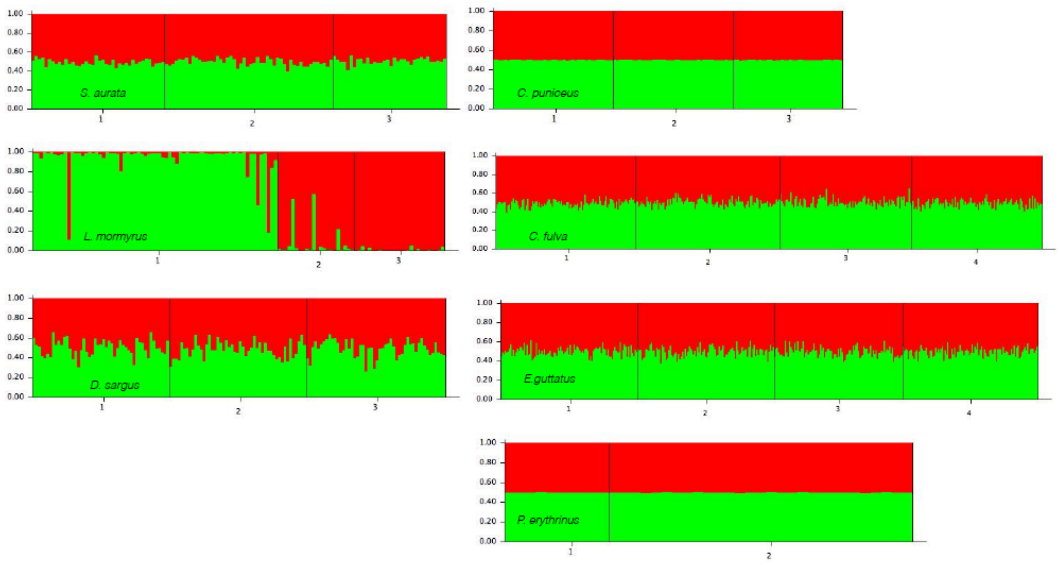

Figure S2. Population structure for each species obtained by STRUCTURE. Admixed individuals in population 1 and 2 (Atlantic and Tyrrhenian respectively) were also removed to provide alternative estimates of N_e, with not significant effects on those estimates.

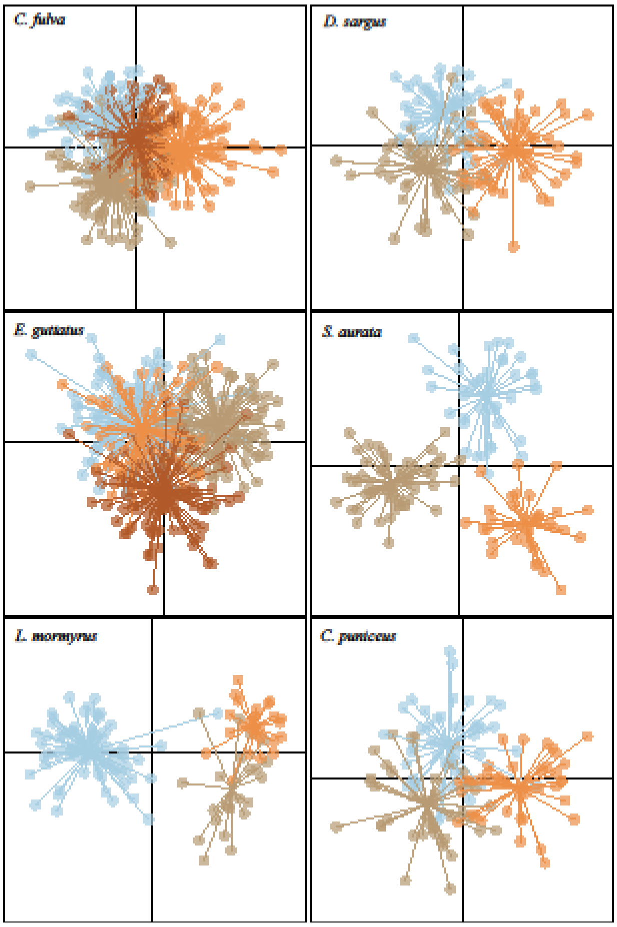

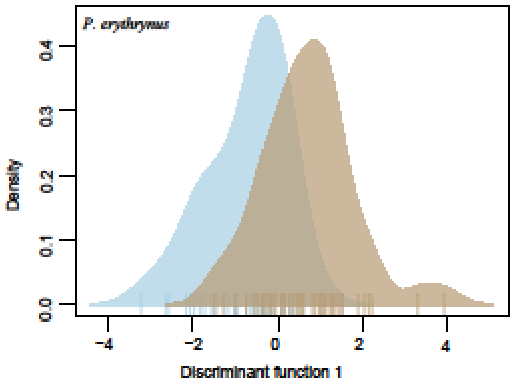

Figure S3. Population structure for each species obtained by Discriminant Analysis of Principal Components (DAPC). Each dot represents an individual and each colour a population. Below, the same analysis for *P. erythrinus*: here the results are not visualised as a scatter, but as a density plot, since there is only one discriminant component to visualise (there are only two populations).

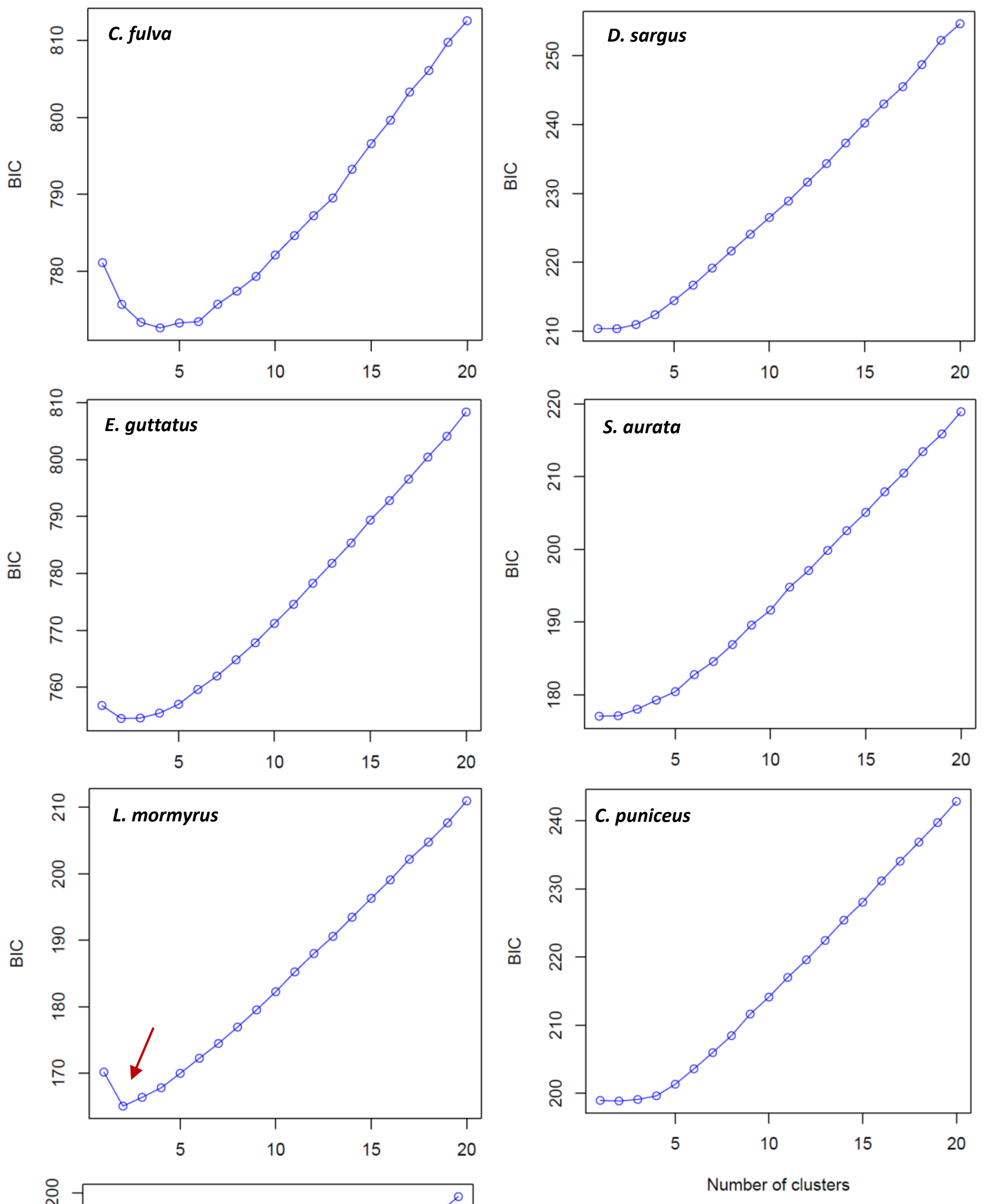

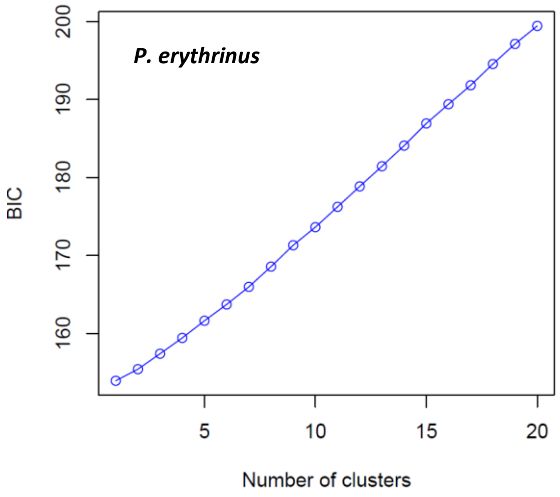

Figure S4. Bayesian information criterion (BIC) for different numbers of clusters of the seven species under investigation (obtained by the function find.clusters in adegenet in R) following Jombart et al. (2010). The red arrow indicates the number of clusters (2) for *L. mormyrus*.

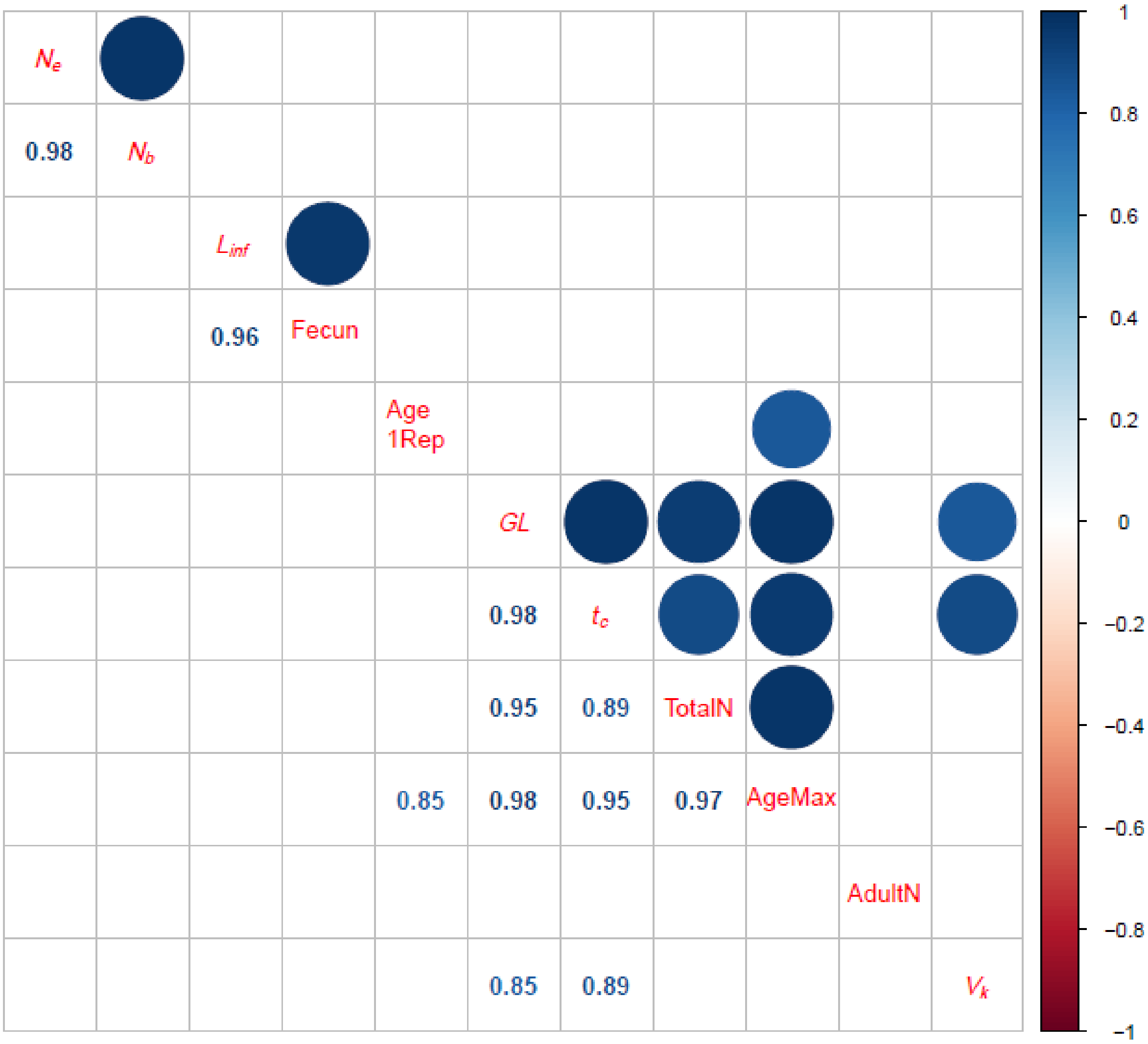

Figure S5. Correlation matrix (Spearman correlation) to check for multicollinearity among variables. Positive correlations are displayed in blue (only significant, $p < 0.01$) in the upper diagonal of the correlogram; size and colour of the circles reflect the values of the correlation coefficient. The corresponding actual correlations values are displayed in the lower diagonal. N_e: effective population size; N_b: number breeders; L_{inf}: maximum length; Fecun: fecundity; Age1Rep: age at first reproduction; *GL*: generation length; t_c: age at sex change; TotalN: estimated number of individuals in the population; AgeMax: maximum age; AdultN: estimated number of adult individuals; V_k: variance in reproductive success.

Table S1. Observed and expected heterozygosity values (H_o and H_e respectively), inbreeding coefficient (F_{is}) and p-value of the Hardy-Weinberg Equilibrium test (HWE) by locus and by population for each species.

	**Ho**			**He**			**Fis**			**HWE**		
D. sargus	Ds1	Ds2	Ds3	Ds1	Ds2	Ds3	Ds1	Ds2	Ds3	Ds1	Ds2	Ds3
DsaMS16	0.88	0.83	0.87	0.95	0.94	0.94	0.07	0.12	0.08	0.31	0.06	0.34
DsaMS27	0.68	0.85	0.78	0.86	0.9	0.91	0.21	0.05	0.14	0.01	0.46	0.06
DsaMS34	0.91	0.85	0.88	0.94	0.95	0.95	0.03	0.1	0.07	0.15	0.04	0.01
DsaMS48	0.83	0.88	0.94	0.89	0.89	0.9	0.06	0.02	-0.04	0.1	0.95	0
Dvul33	0.84	0.86	0.85	0.85	0.86	0.85	0.01	0	0	0.29	0.96	0.53
Dvul4	0.93	0.81	0.83	0.91	0.91	0.92	-0.02	0.11	0.1	0.32	0.17	0.11
Dvul84	0.83	0.76	0.76	0.84	0.86	0.83	0.01	0.12	0.08	0.07	0.18	0.1
Omel38	0.67	0.78	0.61	0.93	0.93	0.9	0.28	0.16	0.33	0	0.03	0
Omel54	0.21	0.24	0.35	0.37	0.27	0.39	0.43	0.13	0.12	0	0.14	0.01
SL26	0.6	0.69	0.9	0.69	0.74	0.71	0.12	0.07	-0.27	0.6	0.57	0.04

	**Ho**			**He**			**Fis**			**HWE**		
L. mormyrus	Lm1	Lm2	Lm3	Lm1	Lm2	Lm3	Lm1	Lm2	Lm3	Lm1	Lm2	Lm3
Ad05LM	0.97	0.73	0.69	0.93	0.81	0.75	-0.05	0.1	0.08	0.4	0.08	0.02
AD66LM	0.9	0.91	0.85	0.92	0.88	0.91	0.02	-0.04	0.07	0.03	0.44	0
Sal15LM	0.99	0.86	1	0.93	0.87	0.9	-0.06	0.01	-0.11	0.03	0.64	0.95
SAI12LM	0.66	0.95	0.96	0.61	0.93	0.9	-0.08	-0.02	-0.07	0.85	0.66	0.96
Bd72LM	0.8	0.91	1	0.87	0.9	0.92	0.08	-0.01	-0.09	0.05	0.26	0.68
SAI19LM	0.91	0.77	0.54	0.91	0.71	0.64	0	-0.1	0.16	0.79	0.96	0.32
Ad86LM	0.94	0.91	0.88	0.94	0.93	0.88	0	0.02	-0.01	0.25	0.77	0.91
Pb102LM	0.89	0.95	0.92	0.91	0.92	0.9	0.03	-0.04	-0.03	0.55	0.67	0.58
Bd68LM	0.8	0.55	0.54	0.79	0.51	0.49	-0.01	-0.07	-0.1	0.04	0.95	0.47

	**Ho**			**He**			**Fis**			**HWE**		
S. aurata	Sa1	Sa2	Sa3	Sa1	Sa2	Sa3	Sa1	Sa2	Sa3	Sa1	Sa2	Sa3
SauD182	0.67	0.74	0.73	0.81	0.8	0.85	0.18	0.07	0.15	0.01	0.03	0.14
SauE82	0.77	0.86	0.76	0.82	0.82	0.82	0.06	-0.04	0.08	0.97	0.77	0.56
SauI47	0.7	0.8	0.7	0.7	0.73	0.78	0	-0.1	0.1	0.19	0.28	0.05
C77b	0.69	0.26	0.5	0.55	0.34	0.6	-0.25	0.23	0.17	0.51	0.09	0.38
Cld.29.T	0.7	0.73	0.9	0.73	0.83	0.8	0.04	0.11	-0.13	0.41	0.14	0.89
Cld.35.H	0.5	0.67	0.7	0.7	0.7	0.76	0.29	0.05	0.08	0	0.08	0.1
Ct27	0.89	0.96	0.95	0.92	0.92	0.94	0.02	-0.05	-0.02	0.02	0	0.03
Fd.92.H	0.95	0.9	0.94	0.87	0.85	0.82	-0.08	-0.05	-0.14	0.92	0.1	0.47
Dd.16	0.77	0.87	0.79	0.88	0.87	0.85	0.13	0.01	0.07	0.1	0	0.58
Dd.84	0.74	0.88	0.71	0.88	0.87	0.81	0.16	0	0.13	0.04	0.77	0.22
Fd.79.T	0.72	0.71	0.68	0.65	0.65	0.63	-0.11	-0.08	-0.08	0.31	0.96	0.8
P20	0.95	0.97	0.92	0.77	0.83	0.76	-0.24	-0.17	-0.2	0	0	0

	Ho			He			Fis			HWE		
C. puniceus	Cp1	Cp2	Cp3	Cp1	Cp2	Cp3	Cp1	Cp2	Cp3	Cp1	Cp2	Cp3
SL1	0.47	0.53	0.54	0.44	0.57	0.45	-0.05	0.06	-0.19	0.35	0.6	0.97
SL17	0.93	1	0.97	0.96	0.97	0.97	0.03	-0.03	-0.01	0.54	0.03	0.46
SL25	0.88	0.84	0.92	0.88	0.82	0.82	0	-0.02	-0.13	0.46	0.26	0.8
SL26	0.77	0.79	0.69	0.7	0.69	0.73	-0.1	-0.15	0.05	0.83	0.33	0.33
SL27	0.91	0.95	0.87	0.98	0.97	0.98	0.07	0.02	0.11	0.03	0	0
SL29	0.63	0.63	0.54	0.62	0.6	0.64	-0.02	-0.05	0.16	0.72	0.51	0.24
SL3	0.95	0.93	0.95	0.95	0.96	0.95	0	0.04	0	0.27	0.16	0.18
SL33	0.91	0.98	0.9	0.91	0.9	0.89	0	-0.09	-0.01	0.1	0.38	0.51
SL34	0.72	0.88	0.82	0.89	0.9	0.9	0.19	0.02	0.09	0.06	0.7	0.32
SL35	0.47	0.44	0.56	0.82	0.81	0.85	0.43	0.45	0.34	0	0	0
SL7	0.98	0.93	0.92	0.96	0.96	0.95	-0.02	0.03	0.03	0.67	0.05	0.03

	Ho		He		Fis		HWE	
P. erythrynus	Pe1	Pe2	Pe1	Pe2	Pe1	Pe2	Pe1	Pe2
ms1	0.58	0.58	0.62	0.7	0.08	0.17	0.55	0.05
ms2	0.18	0.23	0.99	0.97	0.82	0.77	0	0
ms3	0.77	0.64	0.87	0.88	0.11	0.27	0.02	0
ms4	0.66	0.82	0.93	0.95	0.29	0.13	0	0.06
ms8	0.69	0.61	0.95	0.96	0.27	0.36	0	0
sl35	0.83	0.76	0.96	0.97	0.14	0.22	0	0
sl33	0.72	0.61	0.67	0.57	-0.07	-0.08	0.04	1
sl26	0.94	0.89	0.92	0.9	-0.02	0.02	0.34	0.87
ms5	0.66	0.83	0.87	0.92	0.24	0.09	0	0.44
sl1	0.44	0.57	0.97	0.97	0.54	0.42	0	0
sl27	0.59	0.49	0.97	0.96	0.38	0.49	0	0

	Ho				He				Fis				HWE		
C. fulva	Cf1	Cf2	Cf3	Cf4	Cf1	Cf2	Cf3	Cf4	Cf1	Cf2	Cf3	Cf4	Cf1	Cf2	Cf3
CA_002	0.98	1	0.97	0.95	0.95	0.96	0.96	0.95	-0.03	-0.05	-0.01	0	0.56	0.2	0.43
CA_004	0.56	0.58	0.64	0.53	0.54	0.6	0.6	0.58	-0.03	0.03	-0.06	0.09	0.92	0.3	0.8
CA_008	0.72	0.74	0.71	0.71	0.76	0.71	0.73	0.71	0.06	-0.03	0.03	0	0.01	0.43	0.42
GATA_034	0.8	0.91	0.85	0.88	0.85	0.86	0.87	0.85	0.06	-0.06	0.03	-0.04	0.26	0.16	0.04
Cfu9	0.03	0.05	0.11	0.05	0.03	0.04	0.11	0.05	-0.01	-0.01	-0.04	-0.01	1	1	1
Cfu10	0	0	0.16	0.01	0	0	0.16	0.01	NA	NA	0.03	0	1	1	0.56
Cfu14	0.02	0.08	0.1	0.05	0.02	0.08	0.11	0.05	0	-0.02	0.13	-0.02	1	1	0.28
Cfu17	0.14	0.05	0.17	0.09	0.13	0.05	0.16	0.09	-0.07	-0.02	-0.07	-0.03	1	1	1
Cfu18	0.25	0.25	0.25	0.2	0.29	0.32	0.32	0.23	0.13	0.2	0.22	0.14	0.27	0.05	0.15
Cfu20	0.06	0.05	0.01	0.05	0.06	0.05	0.01	0.05	-0.02	0.15	0	-0.01	1	0.02	1
Cfu21	0.89	0.89	0.91	0.93	0.92	0.93	0.92	0.92	0.03	0.04	0.01	-0.01	0.18	0.18	0.35
Cfu23	0.71	0.64	0.75	0.61	0.67	0.6	0.69	0.62	-0.06	-0.07	-0.09	0.02	0.85	0.14	0.34
Cfu25	0.69	0.7	0.76	0.74	0.67	0.68	0.69	0.7	-0.03	-0.03	-0.1	-0.06	0.2	0.65	0.51
Cfu26	0.63	0.7	0.66	0.62	0.71	0.76	0.74	0.71	0.1	0.08	0.11	0.13	0.19	0.14	0.34
Cfu43	0.44	0.55	0.5	0.47	0.42	0.55	0.48	0.44	-0.06	-0.01	-0.03	-0.08	0.67	0.07	0.63
Cfu46	0.03	0.07	0.02	0.03	0.05	0.07	0.02	0.03	0.39	-0.03	-0.01	-0.01	0.05	1	1
Cfu52	0.79	0.82	0.83	0.84	0.8	0.8	0.78	0.82	0.02	-0.02	-0.06	-0.03	0.66	0.97	0.08
Cfu57	0.8	0.84	0.81	0.81	0.78	0.79	0.77	0.76	-0.03	-0.06	-0.06	-0.07	0.67	0.24	0.81
Cfu69	0.42	0.55	0.38	0.47	0.45	0.5	0.42	0.49	0.05	-0.1	0.09	0.05	0.76	0.96	0.25
Cfu70	0.65	0.65	0.68	0.73	0.71	0.7	0.74	0.73	0.09	0.08	0.09	0.01	0.44	0.17	0.01
Cfu72	0.85	0.88	0.94	0.88	0.89	0.91	0.91	0.9	0.05	0.03	-0.04	0.02	0.33	0.14	0.94
Cfu75	0.92	0.85	0.88	0.85	0.9	0.88	0.89	0.9	-0.02	0.03	0.01	0.06	0.76	0.1	0.43
Cfu80	0.68	0.7	0.58	0.74	0.68	0.69	0.65	0.67	0	-0.02	0.11	-0.1	0.17	0.92	0.54
Cfu82	0.32	0.36	0.44	0.39	0.28	0.35	0.47	0.38	-0.13	-0.04	0.06	-0.04	0.4	1	0.67

	Ho				He				Fis				HWE			
E. guttatus	Eg1	Eg2	Eg3	Eg4	Eg1	Eg2	Eg3	Eg4	Eg1	Eg2	Eg3	Eg4	Eg1	Eg2	Eg3	Eg4
CA_002	0.93	0.91	0.96	0.96	0.9	0.91	0.9	0.91	-0.03	0	-0.06	-0.06	0.78	0.31	0.25	0.13
CA_004	0.85	0.91	0.89	0.89	0.89	0.91	0.89	0.91	0.05	0	0	0.02	0.53	0.22	0.53	0.24
CA_008	0.96	0.94	0.98	0.97	0.94	0.95	0.94	0.94	-0.02	0.01	-0.04	-0.03	0.21	0.36	0.72	0.77
GATA_002	0.98	0.91	0.95	0.98	0.97	0.97	0.97	0.97	-0.01	0.06	0.02	-0.01	0.03	0.05	0.2	0.45
GATA_003	0.74	0.69	0.81	0.68	0.75	0.74	0.75	0.72	0.02	0.07	-0.08	0.05	0.96	0.8	0.5	0.17
GATA_015	0.8	0.82	0.81	0.88	0.88	0.89	0.89	0.88	0.09	0.08	0.09	0	0.14	0.59	0	0.57
GATA_018	0.89	0.9	0.93	0.94	0.95	0.94	0.95	0.95	0.06	0.04	0.02	0.01	0.68	0.11	0.02	0.34
GATA_023	0.91	0.89	0.89	0.87	0.91	0.91	0.91	0.92	0	0.02	0.02	0.05	0.66	0.51	0.16	0.32
GATA_034	0.86	0.92	0.91	0.87	0.89	0.88	0.89	0.88	0.04	-0.04	-0.01	0.01	0.12	1	0.24	0.9
GATA_057	0.89	0.92	0.95	0.9	0.9	0.88	0.89	0.89	0.01	-0.04	-0.06	-0.01	0.74	0.8	0.1	0.63
GATA_065	0.83	0.89	0.82	0.86	0.85	0.87	0.86	0.86	0.02	-0.02	0.04	0	0.71	0.39	0.16	0.96
Cfu9	0	0.17	0.04	0.1	0	0.16	0.04	0.1	NA	-0.08	-0.02	-0.05	1	1	1	1
Cfu10	0.13	0.12	0.12	0.13	0.12	0.11	0.11	0.14	-0.04	-0.04	-0.05	0.09	1	1	1	0.32
Cfu14	0.32	0.19	0.35	0.23	0.29	0.22	0.33	0.24	-0.09	0.13	-0.07	0.04	0.67	0.25	0.92	0.08
Cfu20	0.92	0.85	0.89	0.85	0.89	0.86	0.88	0.87	-0.03	0.01	-0.02	0.03	0.04	0.9	0.2	0.64
Cfu25	0.03	0.05	0.05	0.01	0.03	0.05	0.05	0.01	-0.01	-0.02	-0.02	0	1	1	1	1
Cfu43	0.33	0.23	0.31	0.35	0.35	0.23	0.32	0.33	0.07	0.01	0.03	-0.06	0.52	0.65	0.11	0.76
Cfu57	0.88	0.92	0.97	0.87	0.93	0.93	0.94	0.92	0.05	0.01	-0.02	0.06	0.18	0.34	0.25	0.5
Cfu80	0.74	0.72	0.69	0.79	0.72	0.73	0.74	0.75	-0.04	0.01	0.07	-0.05	0.52	0.38	0.25	0.42

Table S2. F_{st} (microsatellite genotypes) values for pairwise comparisons of different populations of the seven species under investigation (lower half of the matrix). In the upper half the correspondent 95% confidence intervals, in brackets. Values in bold reflect statistically significant comparisons.

E. guttatus	Eg1	Eg2	Eg3	Eg4
Eg1		(-0.0004-0.0044)	(-0.0029-0.0011)	(-0.0017-0.0030)
Eg2	0.0017		(-0.0010-0.0036)	(-0.0014-0.0032)
Eg3	-0.001	0.0012		(-0.0017-0.0024)
Eg4	5.00E-04	7.00E-04	2.00E-04	

C. fulva	Cf1	Cf2	Cf3	Cf4
Cf1		(-0.0012-.0057)	**(0.0020-0.0096)**	(-0.0028-0.0020)
Cf2	0.0018		**(0.0007-0.0074)**	(-0.0019-0.0041)
Cf3	**0.0054**	**0.0037**		(-0.0008-0.0060)
Cf4	-5.00E-04	6.00E-04	2.50E-03	

D. sargus	Ds1	Ds2	Ds3
Ds1		(-0.0371-0.0474)	(-0.0039-0.0877)
Ds2	-0.001		(-0.0166-0.0487)
Ds3	0.0051	0.0019	

C. puniceus	Cp1	Cp2	Cp3
Cp1		(-0.0067-0.0104)	(-0.0061-0.0088)
Cp2	9.00E-04		(-0.0047-0.0114)
Cp3	5.00E-04	0.002	

L. mormyrus	Lm1	Lm2	Lm3	Lm4	Lm5
Lm1		**(0.0025-0.0145)**	**(0.0211-0.0381)**	**(0.0177-0.0430)**	**(0.0319-0.0583)**
Lm2	**0.0079**		**(0.0577-0.0759)**	**(0.0496-0.0777)**	**(0.0693-0.0960)**
Lm3	**0.0294**	**0.0664**		**(0.0016-0.0216)**	**(0.0042-0.0202)**
Lm4	**0.0302**	**0.064**	**0.0104**		(-0.0026-0.0309)
Lm5	**0.0430**	**0.0804**	**0.0112**	0.0125	

S. aurata	Sa1	Sa2	Sa3
Sa1		**(0.0038-0.0261)**	(-0.0005-0.0210)
Sa2	**0.0131**		(-0.0043-0.0197)
Sa3	0.009	0.0061	

P. erythrinus	Pe2
Pe1	-0.0011

Table S3. Comparison of known mortality and fecundity rates from literature with average mortality data (across all size classes) calculated with different methods and range of fecundity; proportion of the terminal sex (*S*; males in protogynous species and females in protandrous species) obtained from literature and calculated for each species.

Species		Fixed Mortality (Fishbase)	Calculated Mortality (Charnov et al., 2013)	Calculated mortality (adjusted for age)	Range of Fecundity	Calculated Range of Fecundity	M:F ratio	Calculated M:F (mean)	Sex_f	Sex_i
C. puniceus	PG	0.35*	0.45	0.57	18,582-144,820[31]	31,391-139,159	1:9.5 [76]	1:7.28	25%	7% M_i
P. erythrinus	PG	0.29*	0.27	0.34		207,167-17,359,010	1:3			
S. cantharus	PG	0.35	0.41	0.53	31,670-554,070[35] (7yrs)	30,792-1,038,601 (580,335 7yrs)	1:3		30%	7%
S. pulcher	PG	0.25	0.23	0.28	35,000-1,686,925[37] (11-12yrs)	33,679-6,648,226 1,819,030(11-12yrs)				
D. sargus	PA	0.28*	0.37	0.48	Not available	86,385-1,902,532	1:2	1:56	66.5%	10% F_i
L. mormyrus	PA	0.47	0.37	0.48	Not available	83,446-779,413	1:2.1[76]			
S. aurata	PA	0.6	0.32	0.43	Not available	383,712-5,884,075				
L. calcarifer	PA	0.27	0.26	0.32	46,000,000[77] (15yrs)	5,142,781-40,360,174 30,528,249(15yrs)				

* with updated L_{inf}

Table S4. Microsatellite loci for the five sparid species under investigation

	C. puniceus	*P. erythrinus*	*S. aurata*	*L. mormyrus*	*D. sargus*
PAGEERY MS1[78]		✓			
PAGEERY MS2[78]		✓			
PAGEERY MS3[78]		✓			
PAGEERY MS4[78]		✓			
PAGEERY MS5[78]		✓			
PAGEERY MS8[78]		✓			
SL1[79]	✓	✓			
SL3[79]	✓				
SL7[79]	✓				
SL17[79]	✓				
SL25[79]	✓				
SL26[79]	✓	✓			✓
SL27[79]	✓	✓			
SL29[79]	✓				
SL33[79]	✓	✓			
SL34[79]	✓				
SL35[79]		✓			
SauD182[80]			✓		
SauE82[80]			✓		
SauI47[80]			✓		
C77b[81]			✓		
Cld-29-T[81]			✓		
Cld-35-H[81]			✓		
Ct27[81]			✓		
Fd-92-H[81]			✓		
Dd-16[81]			✓		
Dd-84[81]			✓		
Fd-79-T[81]			✓		
P20[81]			✓		
Ad-10*[81]			✓		
Bld-10-T*[81]			✓		
Ed-02**[81]			✓		
Ad05[81]				✓	
Ad66[81]				✓	
SaL1[82]				✓	
Pb-OVI-D102[83]				✓	
LM12[84]				✓	
LM86[84]				✓	
LM68[84]				✓	
LM72[84]				✓	
LM19[84]				✓	
DsaMS16[85]					✓
DsaMS27[85]					✓
DsaMS34[85]					✓
DsaMS48[85]					✓
Dvul33[86]					✓
Dvul4[86]					✓
Dvul84[86]					✓
Omel38[87]					✓
Omel54[87]					✓
Omel58[87]					✓
	10	11	15	9	11

* possibly under directional selection; ** possibly under balancing selection.

References not in the main text

76 Mariani, S., Sala-Bozano, M., Chopelet, J. & Benvenuto, C. Spatial and temporal patterns of size-at-sex-change in two exploited coastal fish. *Environ. Biol. Fishes* **96**(4), 535-541 (2013).

77 Davis, T. L. O. Estimation of fecundity in barramundi, *Lates calcarifer* (Bloch), using an automatic particle counter. *Aust. J. Mar. Freshw. Res.* **35**(1), 111-118 (1984).

78 Ramšak, A., Garoia, F., Guarniero, I., Mannini, P. & Tinti, F. Novel polymorphic microsatellite markers for the common pandora (*Pagellus erythrinus*). *Mol. Ecol. Notes* **3**, 553-555 (2003).

79 Chopelet, J., Helyar, S., Mann, B. & Mariani, S. Novel polymorphic microsatellite loci for the protogynous hermaphrodite slinger sea bream (*Chrysoblephus puniceus*, Sparidae). *Mol Ecol. Resour.* **9**, 1223-1226 (2009).

80 Launey, S., Krieg, F., Haffray, P., Bruant, J. S., Vannier, A. & Guyomard, R. Twelve new microsatellite markers for gilted seabream (*Sparus aurata* L.): characterization, polymorphism and linkage. *Mol. Ecol. Notes* **3**, 457-459 (2003).

81 Franch, R., Louro, B., Tsalavouta, M., Chatziplis, D., Tsigenopoulos, C. S., Sarropoulou, E., Antonello, J., Magoulas, A., Mylonas, C. C., Babbucci, M., Patarnello, T., Power, D. M., Kotoulas, G. & Bargelloni, L. A genetic linkage map of the hermaphrodite teleost fish *Sparus aurata* L. *Genetics* **174**, 851-861 (2006).

82 Brown, R. C., Tsalavouta, M., Terzoglou, V., Magoulas, A. & McAndrew, B. Additional microsatellites for *Sparus aurata* and cross-species amplification within the Sparidae family. *Mol. Ecol. Notes* **5**, 605-607 (2005).

83 Piñera, J. A., Bernardo, D., Blanco, G., Vázquez, E. & Sánchez, J. Isolation and characterization of polymorphic microsatellite markers in *Pagellus bogaraveo*, and cross-species amplification in *Sparus aurata* and *Dicentrarchus labrax*. *Mol. Ecol. Notes* **6**, 33-35 (2006).

84 Sala-Bozano, M., Tsalavouta, M. & Mariani, S. Isolation and characterisation of new polymorphic microsatellite markers for the striped sea bream (*Lithognathus mormyrus*). *Conserv. Genet.* **10**, 1507-1509 (2009).

85 Pérez, L., Infante, C., Ponce, M., Crespo, A., Zuasti, E., Funes, V., Catanese, G. & Manchado, M. Characterization of eight microsatellite markers in the white sea bream, *Diplodus sargus* (Teleostei, Sparidae). *Mol. Ecol. Resour.* **8**, 1291-1293 (2008).

86 Roques, S., Galarza, J. A., Macpherson, E., Turner, G. F. & Rico, C. Isolation and characterization of nine polymorphic microsatellite markers in the two-banded sea bream (*Diplodus vulgaris*) and cross-species amplification in the white sea bream (*Diplodus sargus*) and the saddled bream (*Oblada melanura*). *Mol. Ecol. Notes* **7**, 661-663 (2007).

87 Roques, S., Galarza, J. A., Macpherson, E., Turner, G. F., Carreras-Carbonell, J., Rico, C. Isolation of eight microsatellites loci from the saddled bream, *Oblada melanura* and cross-species amplification in two sea bream species of the genus *Diplodus*. *Conserv. Genet.* **8**, 1255-1257 (2007).
